# Nasopharyngeal carcinoma with paranasal sinus invasion: the prognostic significance and the evidence-based study basis of its T-staging category according to the AJCC staging system

**DOI:** 10.1186/1471-2407-14-832

**Published:** 2014-11-18

**Authors:** Li Tian, Yi-Zhuo Li, Yun-Xian Mo, Li-Zhi Liu, Chuan-Miao Xie, Xue-Xia Liang, Xiao Gong, Wei Fan

**Affiliations:** Imaging Diagnosis and Interventional Center, Sun Yat-sen University Cancer Center; State Key Laboratory of Oncology in South China; Collaborative Innovation Center for Cancer Medicine, 651 Dongfeng Road East, Guangzhou, 510060 People’s Republic of China; Department of Radiation Oncology, Sun Yat-sen University Cancer Center; State Key Laboratory of Oncology in South China; Collaborative Innovation Center for Cancer Medicine, 651 Dongfeng Road East, Guangzhou, 510060 People’s Republic of China; Department of Medical Statistics and Epidemiology, School of Public Health, Sun Yat-sen University, 74 Zhongshan Road Second, Guangzhou, 510080 People’s Republic of China

**Keywords:** Nasopharyngeal carcinoma, Paranasal sinus, Invasion, Prognosis, Staging

## Abstract

**Background:**

To evaluate the prognostic significance of paranasal sinus invasion for patients with NPC and to provide empirical proofs for the T-staging category of paranasal sinus invasion according to the AJCC staging system for nasopharyngeal carcinoma.

**Methods:**

The clinical records and imaging studies of 770 consecutive patients with newly diagnosed, untreated, and nondisseminated NPC were reviewed retrospectively. The overall survival, distant metastasis-free survival, and local relapse-free survival of these patients were analyzed using the Kaplan-Meier method, and the differences were compared using the log-rank test.

**Results:**

The incidence of paranasal sinus invasion was 23.6%, with the rate of incidence of sphenoid sinus invasion being the highest. By multivariate analysis, paranasal sinus invasion was shown to be an independent prognostic factor for overall survival, distant metastasis-free survival, and local relapse-free survival (*p* < 0.05 for all). No significant differences in overall survival, distant metastasis-free survival, and local relapse-free survival were observed between patients with sphenoid sinus invasion alone and those with maxillary sinus and ethmoid sinus invasion (*p* = 0.87, *p* = 0.80, and *p* = 0.37, respectively). The overall survival, distant metastasis-free survival, and local relapse-free survival for patients with stage T3 disease with paranasal sinus invasion were similar to the survival rates for patients with stage T3 disease without paranasal sinus invasion (*p* = 0.22, *p* = 0.15, and *p* = 0.93, respectively). However, the rates of overall survival and local relapse-free survival were better for patients with stage T3 disease with paranasal sinus invasion than for patients with stage T4 disease (*p* < 0.01, and *p* = 0.03, respectively).

**Conclusions:**

Paranasal sinus invasion is an independent negative prognostic factor for NPC, regardless of which sinus is involved. Our results confirm that it is scientific and reasonable for the AJCC staging system for nasopharyngeal carcinoma to define paranasal sinus invasion as stage T3 disease.

## Background

Nasopharyngeal carcinoma (NPC) is endemic in Southeast Asia, especially in the southern provinces of China [1]. The occurrence of paranasal sinus invasion is not unusual, with an incidence of nearly 30% based on CT and MRI findings [2]. Sphenoid sinus invasion is the most common, followed by maxillary sinus and ethmoid sinus invasion.

The tumor-node-metastasis (TNM) staging system for malignancies is used to evaluate prognosis, aid treatment planning, and facilitate the stratification of treatment. At present, the seventh edition of the American Joint Committee on Cancer (AJCC) staging system is widely used throughout the world, and patients with NPC and paranasal sinus invasion are defined as stage T3 according to the staging system [3]. With regard to the prognostic value of paranasal sinus invasion and its suitable position in the T staging, there are few literature reports for reference. Tao et al. developed a prognostic scoring system (PSS) that could help identify NPC patients with different risk for locoregional relapse, and found that sphenoid sinus, ethmoid sinus and maxillary sinus invasion were classified as different risk groups [4]. While Mao et al. considered sphenoidal sinus invasion alone had a better outcome for patients with NPC than did other paranasal sinus invasion [5]. Both studies indicated that tumor invasion into the different paranasal sinuses might have different effects on the prognosis of patients with NPC. On the other hand, the results of Pan et al. revealed that when paranasal sinus invasion were classified as T3 according the 7th edition AJCC T classification, the segregation of LRFS curves between stage T3 and T4 groups could be well displayed [6]. Which, in a sense, have provided evidence and reference for the AJCC T- staging.

In the present staging system for NPC, radiologic imaging, especially MRI, plays an important role. In comparison to CT, MRI, with its superior soft-tissue contrast, can provide a more accurate definition of early invasion beyond the nasopharynx and a more accurate assessment of the parapharyngeal space, skull base, paranasal sinus, and cranial nerve invasion [7–9]. Given these advantages, MRI is considered the optimal imaging technique for studying the extension of local disease in NPC.

Therefore, we conducted a retrospective study with a large sample size to evaluate the prognostic significance of paranasal sinus invasion for patients with NPC and its suitable position in the T classification, and thus to provide more empirical proofs for the AJCC staging system.

## Methods

### Patient population

This retrospective study was approved by the Institutional Review Board of Sun Yat-Sen University, Guangzhou, China. Between December 2003 and December 2005, 782 consecutive patients with newly diagnosed, untreated, and nondisseminated NPC were recruited for this study. Of the 782 patients, 12 were subsequently eliminated from the study, including nine patients who were unable to complete radiation therapy and three patients in whom new pulmonary nodules and hepatic lesion were detected when the first course of treatment just started. The remaining 770 patients were included in our retrospective study. The median age of the patients was 44 years (range, 13–75 years), with a male-to-female ratio of 3.3:1. All of the patients underwent a pretreatment evaluation that included a complete patient history, physical and neurologic examinations, hematologic and biochemistry profiles, *whole* MR imaging of the neck and nasopharynx, chest radiography, and abdominal ultrasonography. A total of 225 patients with stage N2 or N3 disease underwent emission computed tomography (ECT), and 32 of the 770 patients (4.2%) underwent positron emission tomography-CT. The patients’ medical records and imaging studies were analyzed retrospectively, and the NPC stage was classified according to the seventh edition of the AJCC staging system [3]. The characteristics of the 770 patients are shown in Table 1.

**Table 1:** **The characteristics of 770 patients with nasopharyngeal carcinoma**

Characteristics	Number of patients(%)
Sex	
Male	590(76.6%)
Female	180(23.4%)
Age	
≥50 years old	255(33.1%)
<50 years old	515(69.9%)
Histologic type	
WHO II/III	755(98.1%)
WHO I	15(1.9%)
T classification	
T1	121(15.7%)
T2	115(14.9%)
T3	346(44.9%)
T4	188(24.5%)
N classification	
N0	83(10.8%)
N1	462(60.0%)
N2	203(26.4%)
N3	22(2.8%)
Stage	
I	120(15.6%)
II	234(30.4%)
III	302(39.2%)
IVA ~ IVB	114(14.8%)

### MR imaging protocol and image assessment

All patients underwent MR imaging with a 1.5-T system (Signa CV/i; GE Healthcare, Chalfont St Giles, England). The region from the suprasellar cistern to the inferior margin at the sternal end of the clavicle was examined with a head-and-neck coil. T1-weighted, fast spin-echo images in the axial, coronal, and sagittal planes (repetition time msec/echo time msec, 500–600/10–20) and T2-weighted, fast spin-echo MR images in the axial plane (4000–6000/95–110) were obtained before the injection of contrast material. After intravenous administration of gadopentetate dimeglumine (Magnevist; Schering, Berlin, Germany) at a dose of 0.1 mmol per kilogram of body weight, the axial and sagittal T1-weighted spin-echo sequences and coronal T1-weighted fat-suppressed spin-echo sequences were performed sequentially using the same parameters applied prior to the injection of gadopentetate dimeglumine. A section thickness of 5 mm, an intersection gap of 1 mm and a matrix of 512 × 512 were used.

**Figure 1:**
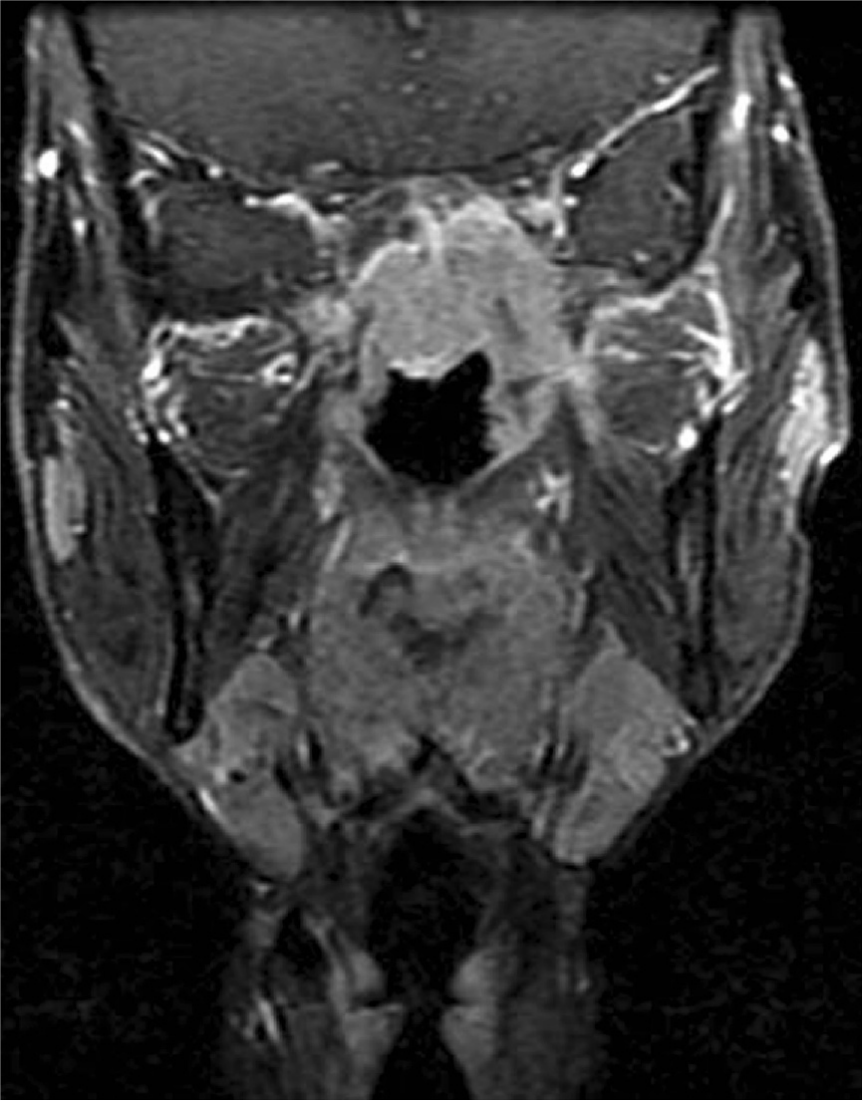
**Image of tumor invasion into the sphenoid sinus in patient with NPC.** A contrast enhanced coronal T1-weighted MR image revealed that the mass connected with the primary nasopharyngeal lesion invaded into the sphenoid sinus and that the floor of the sphenoid sinus was destroyed.

**Figure 2:**
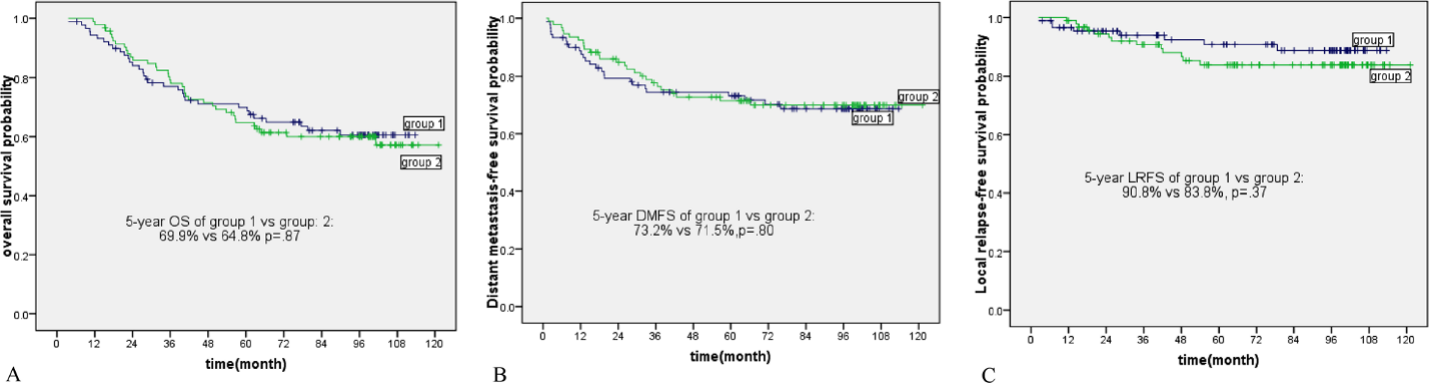
**Survival curves of patients with NPC and different paranasal sinus invasion.** The graph shows **(A)** the overall survival probability, **(B)** the distant metastasis-free survival probability, and **(C)** the local relapse-free survival probability for patients with sphenoid sinus invasion alone and patients with maxillary sinus and ethmoid sinus invasion. Group 1 and Group 2 represent patients with NPC with sphenoid sinus invasion alone and with maxillary sinus and ethmoid sinus invasion, respectively.

All MR images were reviewed by two radiologists with more than 10 years of experience in MR imaging of head and neck cancers. All images were evaluated independently, and disagreements were resolved by consensus. Diagnostic MRI criteria for the invasion of the paranasal sinuses included the following: (1) tumors that had invaded into the sinus cavity connected with a primary nasopharyngeal lesion and with bone destruction of the wall of the sinus (Figure 1) and (2) presentation with an equal or lower signal in the T1WI MRI scan, an equal or higher signal in the T2WI and an obvious enhancement in the enhanced MRI scan, with the same signal intensity characteristics as revealed in the primary lesion [2, 10].

### Treatment

All patients were treated with definitive-intent radiation therapy. A total of 618 of the 770 patients (80.2%) underwent two-dimensional conventional radiation therapy, 115 (14.9%) underwent intensity-modulated radiation therapy (IMRT), and 37 (4.8%) underwent three-dimensional conformal radiation therapy. Details regarding the radiation therapy techniques have been reported previously [11–13].

Of the 416 patients with stage III or IV NPC (classified as stage T3-T4 and/or stage N2-N3), 370 (89%) received neoadjuvant, concomitant, or adjuvant chemotherapy. When possible, salvage treatments, including afterloading, surgery, and chemotherapy, were provided in the event of documented relapse or if the disease persisted.

### Follow up

The follow-up period was estimated from the first day of treatment to either the day of death or the day of the last examination. Follow up was performed with imaging or clinical assessment. The patients were evaluated at least once every three months during the first two years; thereafter, patients were followed up every six months until death.

### Statistical analysis

The Statistical Package for Social Sciences 15.0 (SPSS, Chicago, IL) was used for statistical analysis. The following endpoints (interval to the first defining event) were assessed: overall survival (OS), distant metastasis-free survival (DMFS), and local relapse-free survival (LRFS).The actual rates were calculated using the Kaplan-Meier method, and the differences were compared with the log-rank test [14]. Multivariate analyses with the Cox proportional hazards model were used to test independent significance by the backward elimination of insignificant explanatory variables [15]. The criterion for statistical significance was set at α = 0.05, and *p* values were based on two-sided test results.

## Results

### Incidence of paranasal sinus invasion

The incidence of paranasal sinus invasion was 23.6% (182 of 770 patients), with invasion of the sphenoid sinus, maxillary sinus and ethmoid sinus in 162 (21.0%), 86 (11.2%) and 38 (4.9%) of the 770 patients, respectively. None of the patients had frontal sinus invasion. The incidence of sphenoid sinus invasion was higher than that of maxillary sinus and ethmoid sinus invasion in patients with NPC. Of the 162 patients with sphenoid sinus invasion, 89 (54.9%) did not have maxillary sinus and ethmoid sinus invasion. In contrast, of the 86 patients with maxillary sinus invasion and 38 patients with ethmoid sinus invasion, 69 (80.2%) and 32 (84.2%) also had sphenoid sinus invasion, respectively. Of the 182 patients with paranasal sinus invasion, 97 (53.3%) had stage T3 disease, and 85 (46.7%) had stage T4 disease.

### Prognosis of patients with paranasal sinus invasion

The median follow-up period was 84 months (range, 3–120 months). In total, 59 patients (7.6%) developed local-regional failure, 129 patients (16.8%) developed distant metastases, and 184 patients (23.9%) died. The 5-year overall survival, distant metastasis-free survival, and local relapse-free survival rates for the entire patient population were 80.2%, 84.7%, and 92.4%, respectively. Significant differences were observed between patients without and with paranasal sinus invasion in overall survival (84.4% vs 67.2%, *P* < 0.01), distant metastasis-free survival (88.4% vs 72.3%, *P* < 0.01) and local relapse-free survival (94.0% vs 87.0%, *P* < 0.01), with better outcomes associated with patients without paranasal sinus invasion.

The following parameters, which could possibly influence the prognosis, were included in the Cox proportional hazards model for multivariate analysis: age (≥50 years and <50 years), sex, nasal cavity extension, oropharyngeal extension, parapharyngeal space extension, skull base erosion, paranasal sinus extension, hypopharyngeal extension, orbit extension, masticator space extension, cranial nerve palsy and intracranial extension, N classification, use of chemotherapy, radiation therapy technique. The N classification was treated as ordinary variable in the multivariate analysis. Using multivariate analysis, paranasal sinus invasion was identified as an independent prognostic factor for overall survival, distant metastasis-free survival, and local relapse-free survival (P < 0.05 for all). The parapharyngeal space extension and N classification were found to be independent prognostic factors for both overall survival and distant metastasis-free survival (Table 2).

**Table 2:** **Multivariate analysis of prognostic factors for patients with nasopharyngeal carcinoma**

Endpoint and Variable	***P Value***	Odds Ratio (95% confidence interval)
Overall survival		
*Age*	*<0.01*	*1.80(1.35,2.42)*
*Paranasal sinus involvement*	*<0.01*	*1.76(1.28.2.42)*
*Parapharyngeal space extension*	*<0.01*	*1.95(1.31,2.90)*
*Intracranial extension*	*<0.01*	*1.74(1.27,2.40)*
*N classification*	*0.02*	*0.69(0.51,0.93)*
Distant metastasis-free survival		
*Age*	*<0.01*	*1.75(1.23,2.48)*
*Paranasal sinus involvement*	*0.02*	*1.58(1.08,2.30)*
*Parapharyngeal space extension*	*0.04*	*1.62(1.02,2.58)*
*Skull base erosion*	*<0.01*	*2.73(1.45,5.16)*
*N classification*	*<0.01*	*0.63(0.44, 0.89)*
Local relapse-free survival		
*Paranasal sinus involvement*	*0.02*	*1.91(1.11,3.27)*

### T-staging category of paranasal sinus invasion

A total of 182 patients developed paranasal sinus invasion. Owing to the proximity of the floor of the sphenoid sinus to the roof of the nasopharynx, and the majority of patients with maxillary sinus or ethmoid sinus invasion accompanied with sphenoid sinus invasion simultaneously, the 182 patients were divided into two groups. Group 1 was composed of patients with invasion of the sphenoid sinus alone, without invasion of the maxillary sinus and ethmoid sinus, and group 2 was composed of patients with invasion of the maxillary sinus and/or ethmoid sinus. Of the 182 patients with paranasal sinus invasion, 89 and 93 had group 1 and group 2 invasion, respectively. No significant differences in overall survival, distant metastasis-free survival and local relapse-free survival were observed between the patients with group 1 and group 2 invasion (*p* = 0.87, *P* = 0.80, and *p* = 0.37, respectively, Figure 2).

**Figure 3:**
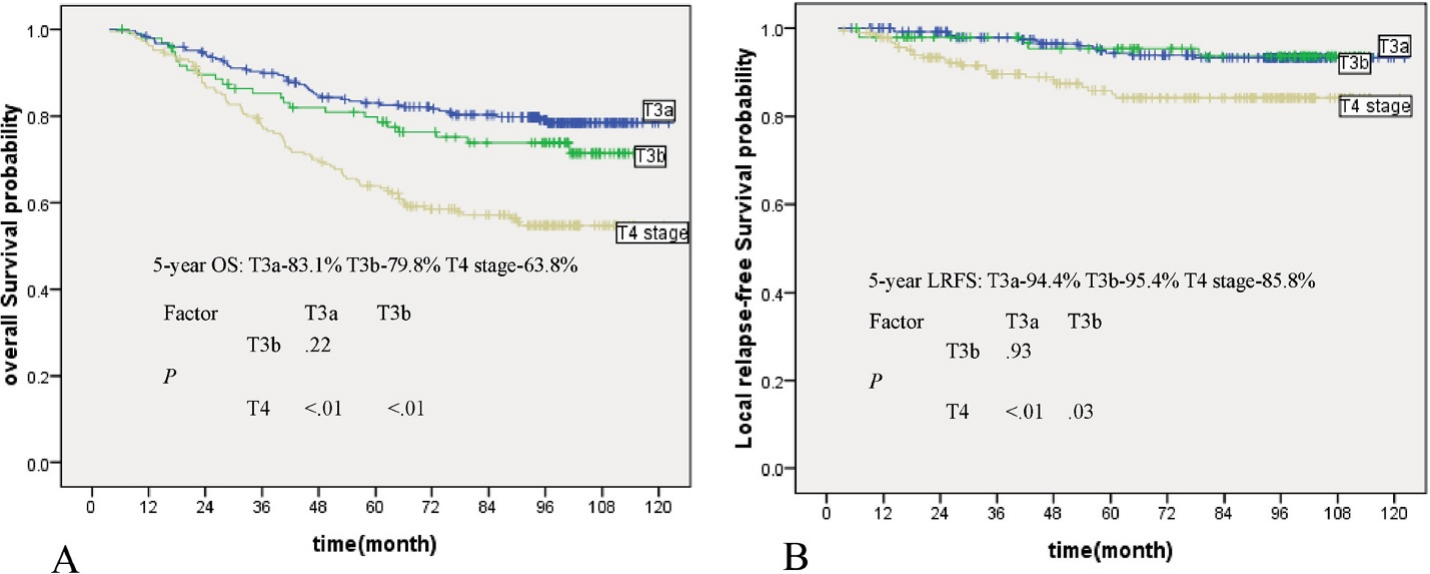
**Survival curves of patients with NPC with T3 and T4 disease.** The graph shows **(A)** the overall survival probability and **(B)** the local relapse-free survival probability for patients with stage T3 and T4 disease according to the seventh AJCC staging system. T3a and T3b represent patients with T3 disease without and with paranasal sinus invasion, respectively.

According to the seventh AJCC staging system, 346 patients belonged to stage T3, of which, 249 did not develop paranasal sinus invasion (T3a) and 97 developed paranasal sinus invasion (T3b). No significant differences in overall survival, distant metastasis-free survival and local relapse-free survival were observed between patients with T3a and those with T3b (p = 0.22, p = 0.15, and p = 0.93, respectively). However, the rates of overall survival and local relapse-free survival were better for patients with T3b than for patients with stage T4 disease (p < 0.01, and p = 0.03, respectively) (Figure 3). No significant difference in distant metastasis-free survival was observed between patients with T3b and those with T4 disease (p = 0.10). When paranasal sinus invasion was classified as stage T3, the segregation of survival curves between the T3 and T4 groups was clearly displayed.

## Discussion

### The incidence of paranasal sinus invasion of patients with NPC

NPC is an aggressive neoplasm, and the spread of the tumor into the paranasal sinuses occurs relatively frequently. The result of the present study, based on the data from a large cohort, suggested that the incidence of the invasion of the paranasal sinus in patients with NPC was 23.6%. The highest rate of incidence was of sphenoid sinus invasion (21%), followed by maxillary sinus invasion (11.2%) and ethmoid sinus invasion (4.9%). Chong et al. reported the CT and MRI findings of 114 patients with NPC, 21%, 9% and 4% of those patients were detected with sphenoid sinus, maxillary sinus and ethmoid sinus invasion, respectively [2]. While the results of King et al. showed that the incidence rates of sphenoid sinus, maxillary sinus and ethmoid sinus invasion were 27%, 5% and 14%, respectively [16]. Our results are roughly the same as those of Chong et al., but a little different from those of King et al. For patients with NPC, the local disease spreads in a stepwise manner from proximal to distal sites [7]. Tumors of the roof of the nasopharynx tend to spread directly and superiorly into the skull base, where there is no muscle or fascia to act as a barrier against tumor invasion, as well as to the floor of the sphenoid sinus, which borders the nasopharynx roof. Therefore, for most patients with NPC, the primary tumor originating from the nasopharynx directly destroys the floor of the sphenoid sinus. This accounts for the high rate of incidence of sphenoid sinus invasion. Additionally, the posterior wall of the maxillary sinus is adjacent to the pterygopalatine fossa and accordingly, tumors extending anteriorly to the pterygopalatine fossa can easily spread to the maxillary sinus. In contrast, tumors extending to the sphenoid sinus or nasal cavity are likely to invade the ethmoid sinus anteriorly or superiorly.

### The prognostic significance of paranasal sinus invasion for patients with NPC

Our retrospective study, based on large number of cases, revealed that the invasion of the paranasal sinus was an independent negative prognostic factor for overall survival, distant metastasis-free survival, and local relapse-free survival in patients with NPC. Hence, it is scientific and reasonable for paranasal sinus invasion to be included in the AJCC staging system. Some researchers proposed that the maximum primary tumor diameter (MPTD) or primary tumor volume (PTV) had some effect on the prognosis of patients with NPC [17–21]. Due to the proximity of the floor of the sphenoid sinus to the roof of the nasopharynx, and the majority of patients with maxillary sinus or ethmoid sinus invasion accompanied with sphenoid sinus invasion simultaneously, we had speculated that the primary tumor volume of patients with sphenoid sinus invasion might be a little smaller than that of patients with ethmoid sinus and maxillary sinus invasion, making for somewhat better prognosis for patients. However, the results showed that the prognostic significances for both groups were not significantly different. We speculate this may be due to the following reasons. First, the primary treatment modality for patients with NPC was radiation therapy. With the aid of MRI, the range of local lesions could be evaluated with greater accuracy and the target and field could be designed more rationally [22]. Additionally, the improved treatment strategies for T3-4 patients with NPC, including the boost technique of two-dimensional radiation therapy, IMRT, and the combination of chemotherapy with radiotherapy, have dramatically improved the treatment outcome with respect to loco-regional control [12, 23, 24]. This may be the reason why no difference was observed in the LRFS between patients with sphenoid sinus invasion alone and those with maxillary sinus and ethmoid sinus invasion. Second, as for the DMFS rate, we think the possible reason may lie in the fact that the difference of the tumor volume resulted from the different paranasal sinus invasion may not be significant enough to lead to significant difference of risks for distant failure. For the reasons given above, it is reasonable to consider paranasal sinus invasion as a single entity in the TNM classification, regardless of which sinus is involved.

### The evidence-based study basis of T-staging category of paranasal sinus invasion

In the fifth edition of the AJCC staging system for NPC, patients with paranasal sinus invasion were defined as T3, and this classification remains in the sixth and the current seventh edition of the AJCC staging system. While paranasal sinus invasion is classified as stage T4 disease according to the Chinese 2008 staging system [25]. Pan et al. compared the predictive value of both staging systems for patients with NPC [6]. The results revealed that for the Chinese 2008 T classification, the 5-year LRFS rates of T3 and T4 groups did not differ significantly, while the rates between both groups were remarkably different according to the 7th edition AJCC T classification. The possible reason lie in that when compared to cranial nerve palsy and intracranial extension, paranasal sinus invasion maybe occur a little bit earlier, accordingly, the tumor volume may be somewhat smaller, which possibly makes for a better prognosis. In a sense, this study demonstrated that it was more suitable for paranasal sinus invasion to be classified as stage T3. Our results, based on a large number of samples, further confirmed this viewpoint, providing more empirical proofs for the rationality of the AJCC T staging.

### The influence of MRI on patients with NPC

All of the patients in the current study were evaluated by MRI. For the lesions of the paranasal sinus in patients with NPC, many inflammatory changes overlapped with the neoplastic process, and it is important to differentiate between the two entities and to define the inflammation-tumor border. MR imaging, especially T2-weighted and contrast enhanced MR, can help us to solve this challenging problem. The inflammatory lesions usually present high signal intensity on T2-weighted image and a thin superficial enhancement after contrast administration, while the tumors reveal relatively lower signal intensity on T2-weighted image and solid enhancement with contrast administration. In addition, most importantly, the signal intensity and enhancement pattern of tumors invading the paranasal sinus are usually in accordance with those of the primary tumor of the nasopharynx [2, 26, 27] *.* Over the past several decades, MRI has been used to assess the extent of NPC more reliably and accurately compared with CT, which has been shown to influence the stage assignment and disease prognosis [28–30].

### Limitations of this study

It should be stressed that because of limited resources, most of patients (80.2%) in this study were treated with conventional radiotherapy technique. Recently, intensity-modulated radiotherapy (IMRT) has gradually replaced two-dimensional conventional radiotherapy as the primary radiotherapy technique for NPC and has been reported to provide encouraging treatment outcome [31–35]. Therefore, the suitability of the staging system of NPC amid the changes in therapeutic methods needs continual assessment. As well, the effect of paranasal sinus invasion on the prognosis and staging of patients with NPC should be further confirmed by clinic studies.

## Conclusion

Paranasal sinus invasion is an independent negative prognostic factor for NPC, regardless of which sinus is involved. It is scientific and reasonable for paranasal sinus invasion to be defined as stage T3 disease, as proposed in the AJCC staging system. Our study provided some empirical proofs for the AJCC T staging.
